# Competence of radiologists in cardiac CT and MR imaging in Europe: insights from the ESCR Registry

**DOI:** 10.1007/s00330-024-10644-4

**Published:** 2024-02-28

**Authors:** Federica Catapano, Lukas Jakob Moser, Marco Francone, Carlo Catalano, Rozemarijn Vliegenthart, Ricardo P. J. Budde, Rodrigo Salgado, Maja Hrabak Paar, Maja Pirnat, Christian Loewe, Konstantin Nikolaou, Michelle C. Williams, Giuseppe Muscogiuri, Luigi Natale, Lukas Lehmkuhl, Malte Maria Sieren, Matthias Gutberlet, Hatem Alkadhi

**Affiliations:** 1 Department of Radiology, IRCCS Humanitas Research Hospital, Rozzano, Milan Italy; 2 Diagnostic and Interventional Radiology, University Hospital Zurich, University of Zurich, Raemistrasse 100, CH-8091 Zurich, Switzerland; 3 Department of Radiological Sciences, Policlinico Umberto I, Sapienza University of Rome, Rome, Italy; 4 Department of Radiology, University of Groningen/University Medical Center Groningen, Groningen, The Netherlands; 5 Department of Radiology and Nuclear Medicine, Erasmus Medical Center, Rotterdam, The Netherlands; 6 Department of Radiology, Antwerp University Hospital & Antwerp University, Holy Heart Lier, Lier, Belgium; 7 Department of Diagnostic and Interventional Radiology, University Hospital Centre Zagreb, Zagreb, Croatia; 8 Radiology Department, University Medical Centre Maribor, Maribor, Slovenia; 9 Division of Cardiovascular and Interventional Radiology, Department of Biomedical Imaging and Image-Guided Therapy, Medical University of Vienna, Vienna, Austria; 10 Department of Diagnostic and Interventional Radiology, University of Tübingen, Tübingen, Germany; 11 British Heart Foundation Centre for Cardiovascular Science, University of Edinburgh, Edinburgh, UK; 12 Department of Radiology, IRCCS Istituto Auxologico Italiano, San Luca Hospital, Milan, Italy; 13 University of Milano-Bicocca, Milan, Italy; 14 Department of Radiological Sciences - Institute of Radiology, Catholic University of Rome, A. Gemelli University Hospital, Rome, Italy; 15 Clinic for Radiology, Heart Center Bad Neustadt a.d. Saale, Bad Neustadt a.d. Saale, Germany; 16 Department of Radiology and Nuclear Medicine, University Hospital Schleswig-Holstein, Ratzeburger Lübeck, Germany; 17 Institute of Interventional Radiology, University Hospital Schleswig-Holstein, Ratzeburger Lübeck, Germany; 18 Department of Diagnostic and Interventional Radiology, University of Leipzig - Heart Centre, Leipzig, Germany

**Keywords:** Registry data, Cardiac diseases, Magnetic resonance imaging, CT angiography, Cardiac imaging techniques

## Abstract

**Rationale:**

To provide an overview of the current status of cardiac multimodality imaging practices in Europe and radiologist involvement using data from the European Society of Cardiovascular Radiology (ESCR) MRCT-registry.

**Materials and methods:**

Numbers on cardiac CT and MRI examinations were extracted from the MRCT-registry of the ESCR, entered between January 2011 and October 2023 (*n* = 432,265). Data collection included the total/annual numbers of examinations, indications, complications, and reporting habits.

**Results:**

Thirty-two countries contributed to the MRCT-registry, including 29 European countries. Between 2011 and 2022, there was a 4.5-fold increase in annually submitted CT examinations, from 3368 to 15,267, and a 3.8-fold increase in MRI examinations, from 3445 to 13,183. The main indications for cardiac CT were suspected coronary artery disease (CAD) (59%) and transcatheter aortic valve replacement planning (21%). The number of patients with intermediate pretest probability who underwent CT for suspected CAD showed an increase from 61% in 2012 to 82% in 2022. The main MRI indications were suspected myocarditis (26%), CAD (21%), and suspected cardiomyopathy (19%). Adverse event rates were very low for CT (0.3%) and MRI (0.7%) examinations. Reporting of CT and MRI examinations was performed mainly by radiologists (respectively 76% and 71%) and, to a lesser degree, in consensus with non-radiologists (19% and 27%, respectively). The remaining examinations (4.9% CT and 1.7% MRI) were reported by non-radiological specialties or in separate readings of radiologists and non-radiologists.

**Conclusions:**

Real-life data on cardiac imaging in Europe using the largest available MRCT-registry demonstrate a considerable increase in examinations over the past years, the vast majority of which are read by radiologists. These findings indicate that radiologists contribute to meeting the increasing demands of competent and effective care in cardiac imaging to a relevant extent.

**Clinical relevance statement:**

The number of cardiac CT and MRI examinations has risen over the past years, and radiologists read the vast majority of these studies as recorded in the MRCT-registry.

**Key Points:**

*• The number of cardiac imaging examinations is constantly increasing*.

*• Radiologists play a central role in providing cardiac CT and MR imaging services to a large volume of patients*.

*• Cardiac CT and MR imaging examinations performed and read by radiologists show a good safety profile*.

**Graphical Abstract:**

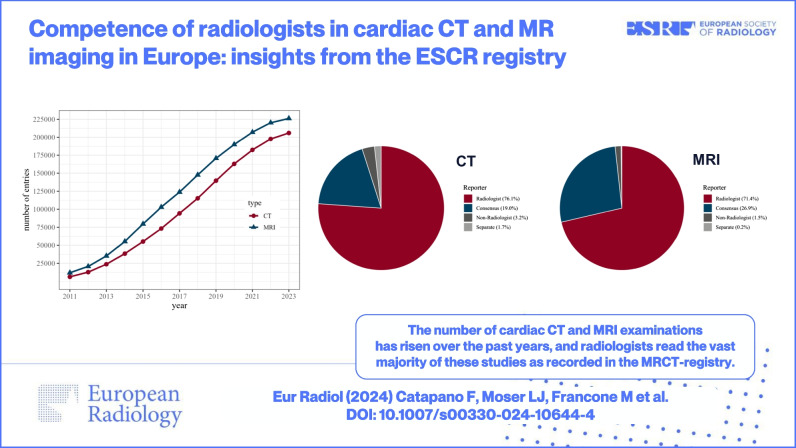

**Supplementary Information:**

The online version contains supplementary material available at 10.1007/s00330-024-10644-4.

## Introduction

Over the past two decades, the indications for advanced cardiovascular imaging have undergone a significant transformation, reshaping diagnostic paradigms in various clinical scenarios. This evolution aligns with the growing body of scientific evidence regarding the central role of computed tomography (CT) and magnetic resonance imaging (MRI) in the diagnosis, prognostic stratification, and therapeutic guidance of cardiovascular disease [1–6].

Coronary CT angiography is recognized as the primary imaging modality for patients with a low-to-intermediate probability of coronary artery disease (CAD) [7]. Moreover, coronary CT angiography has an important role in pre-procedural imaging, spanning from transcatheter aortic valve replacement (TAVR) planning [8, 9] to newer applications in guiding interventional procedures [10].

Stress MRI has shown the overall highest accuracy in diagnosing ischemia, outperforming stress echocardiography and single-photon emission CT, particularly in patients with intermediate to high probability of CAD [11, 12]. The role of cardiac MRI extends beyond CAD risk stratification and myocardial viability assessment. Major consensus documents and guidelines [13–15] advocate its use for a diverse spectrum of clinical conditions including congenital heart disease, cardiomyopathy, myocarditis and its differentials, and when echocardiography yields suboptimal or inconclusive results [16].

Therefore, the demand for multimodality non-invasive cardiovascular imaging is constantly increasing and requires trained professionals [17, 18]. There are some data (in particular for coronary CT angiography) that the anticipated future workload expansion of cardiovascular imaging could outstrip the existing scanner capacity and trained workforce with regard to radiology technicians and imaging specialists [18]. In response to, and in anticipation of this demand, cardiac CT and MRI are now a formal and integral part of every radiology residency training program in Europe and its individual countries, and fellowship programs assist in the training of radiologists subspecialising in multimodality cardiac imaging [19–22].

In 2011, a registry for cardiac CT and MRI examinations, called the *MRCT-registry*, was established under the heading of the European Society of Cardiovascular Radiology (ESCR). This registry serves various purposes, such as to map activity of European cardiac radiology practices including trends in protocols, medication, and indications over time, documenting information for accreditation, identification of expert centres, fostering collaboration for multicentre trials, and ensuring consistent, high-quality patient care.

The current study aims to provide an overview of the status of advanced cardiac imaging practices in Europe and radiologist involvement using the structured database of from the MRCT-registry.

## Methods

### Registry design

The MRCT-registry was established in 2011, the same year that the ESR/ESCR and the German Roentgen Society (DRG) launched cardiovascular imaging certification initiatives, which includes the European Board of Cardiovascular Radiology (EBCR) diploma. The registry was intended to collect anonymized data on the use and indications for cardiac CT and MRI studies in Europe. It remains the only cardiac imaging registry that collects data from both imaging modalities. Complying with the Declaration of Helsinki, a local ethics committee approved the research protocol and waived the need for patient informed consent (Leipzig University; No. 131/17-ek).

### Registry composition and available data

The MRCT-registry incorporates a variety of information, including basic patient characteristics like sex and age, but also details regarding the indication, final diagnosis, and imaging protocol characteristics. Documentation of cardiac CT and MRI cases in the MRCT-registry was a prerequisite to apply for Q1-Q3 certificates of the German Roentgen Society (DRG) [22]. Some previous studies reported results from the MRCT-registry on different aspects [22–25].

We obtained data from the MRCT-registry regarding the number of cardiac CT and MRI examinations that were submitted and their main indication, entered from January 2011 until October 1, 2023. We also extracted information about medications administered prior to or during exams, as well as the number and type of complications, and the reporting physician(s) of cardiac CT and MRI examinations. Possible categories for the latter were (i) reporting by radiologists, (ii) consensus reading (radiologist and non-radiologist), (iii) reporting by a non-radiologist, and (iv) separate readings by radiologist and non-radiologist. Pretest probability for patients with suspected CAD was classified as previously suggested [26]. Adverse events categories were predefined by the design of the registry. The Appendix (Supplementary Material, Table S1) contains the complete list of parameters extracted from the registry for this study.

### Statistical analysis

The objectives of the registry are descriptive in nature; therefore, absolute numbers and percentages were used to describe the registry data. Linear regression models were fit to plot the trends for the indication and pretest probability of CAD in CT and for the indication of myocarditis in MRI, as main indications for CT and MRI, respectively. From the models, 95% confidence intervals were derived and then plotted with the package ggplot2. Fisher’s exact test was used to compare the frequency of adverse events between stress and non-stress MRI examinations. Analysis was performed in R (version 4.3.1, The R Foundation).

## Results

### Contributing countries and number of examinations in the MRCT-registry

**Fig. 1:**
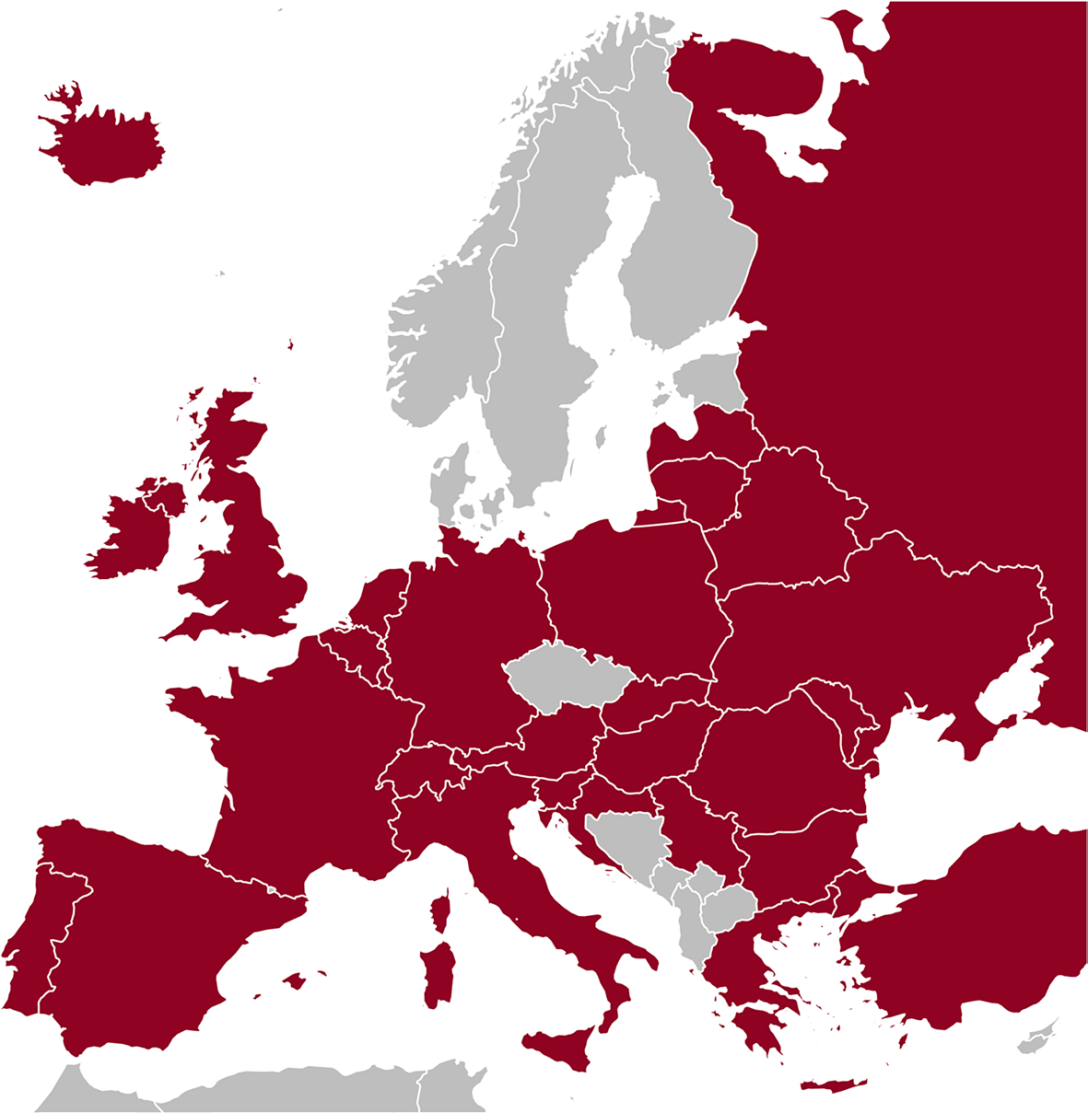
European countries contributing to the MRCT-registry (indicated in red)

**Fig. 2:**
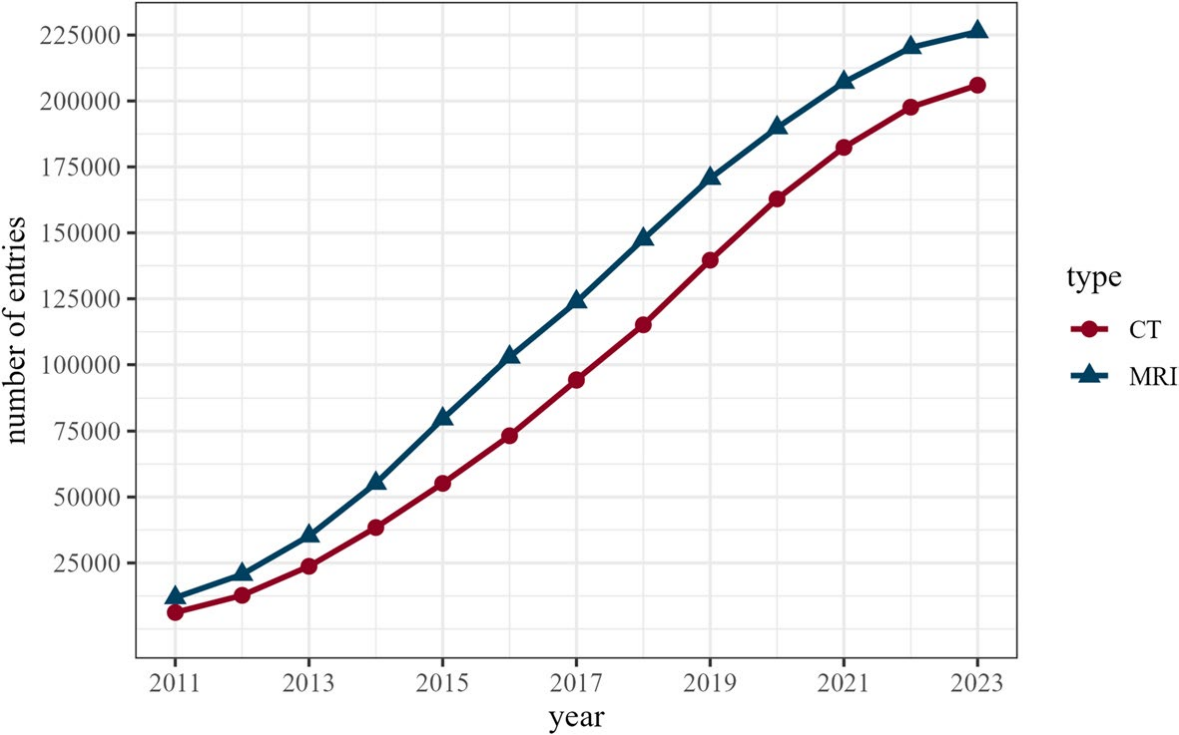
Total number of entries in the ESCR MRCT-registry from January 2011 until October 1, 2023

**Fig. 3:**
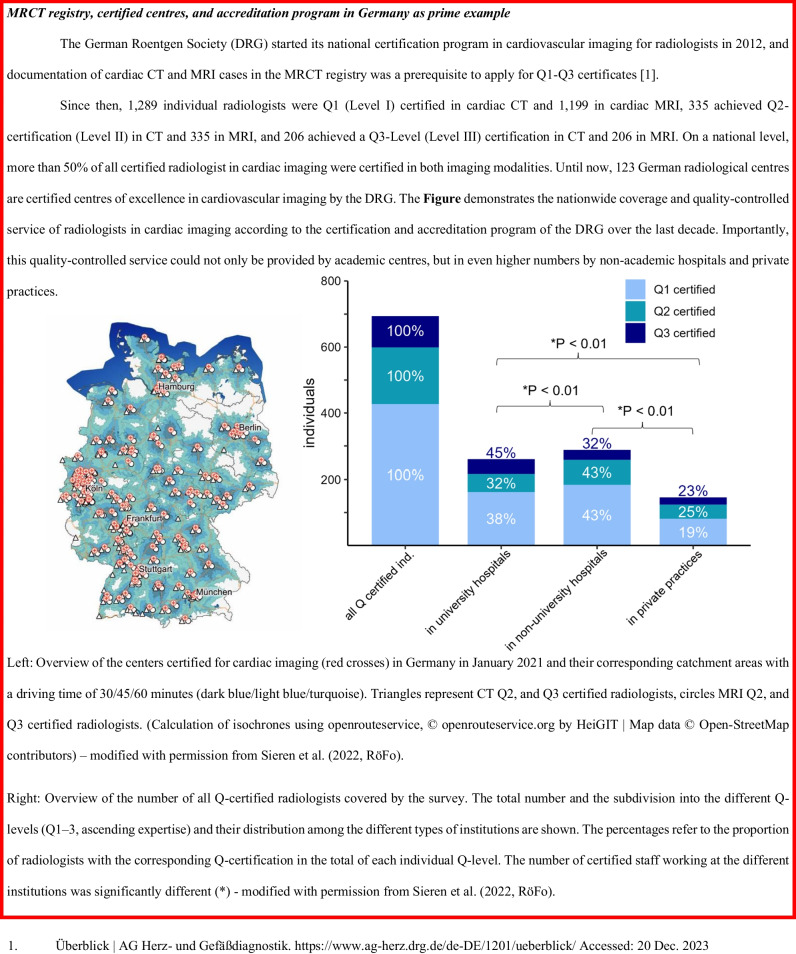
MRCT registry, certified centres, and accreditation program in Germany as prime example

A total of 32 countries contributed to the MRCT-registry, including 29 European countries (Fig. 1). The three participating non-European countries were Bangladesh, Canada, and Kazakhstan. As of October 1, 2023, the ESCR Registry includes 205,999 entries for CT and 226,266 entries for MRI, totalling 432,265 cardiac cross-sectional imaging examinations. Figure 2 shows the trend in submissions from January 2011 to October 2023. Between 2011 and the end of 2022 (the last complete year included in this study), there was a 4.5-fold increase in annually submitted CT examinations, from 3368 to 15,267, and a 3.8-fold increase in MRI examinations, from 3445 to 13,183. The majority (56.8%) of the 1204 individual examiners in the MRCT-registry submitted both cardiac CT and MRI cases, whereas a minority submitted cases in only cardiac CT (22.3%) or MRI (20.8%). The national situation in Germany as a prime example for the MRCT-registry, certification centres, and accreditation program is provided in Fig. 3.

### Main indications

**Fig. 4:**
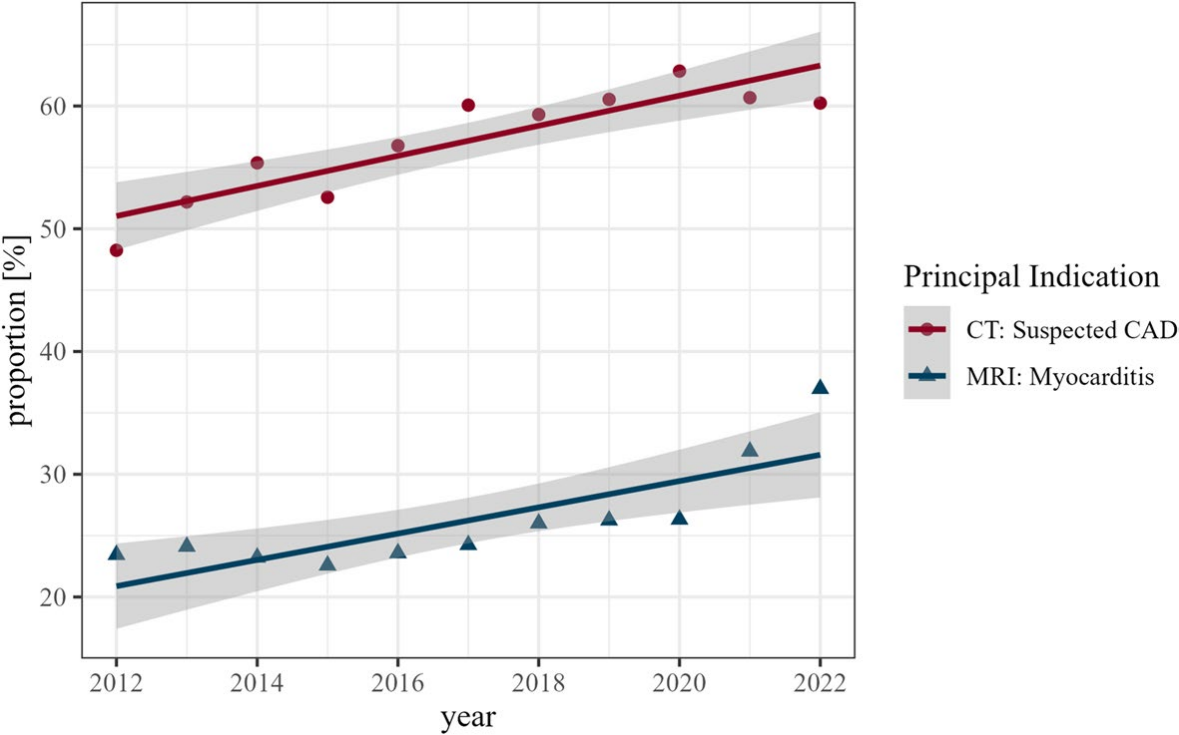
Annual submissions of the principal indication in cardiac CT (red dots) and cardiac MRI (blue triangles). Symbols denote the relative frequency in percentages. Lines represent linear models representing the trend. Grey shading represents the 95% confidence interval derived from the linear models

**Fig. 5:**
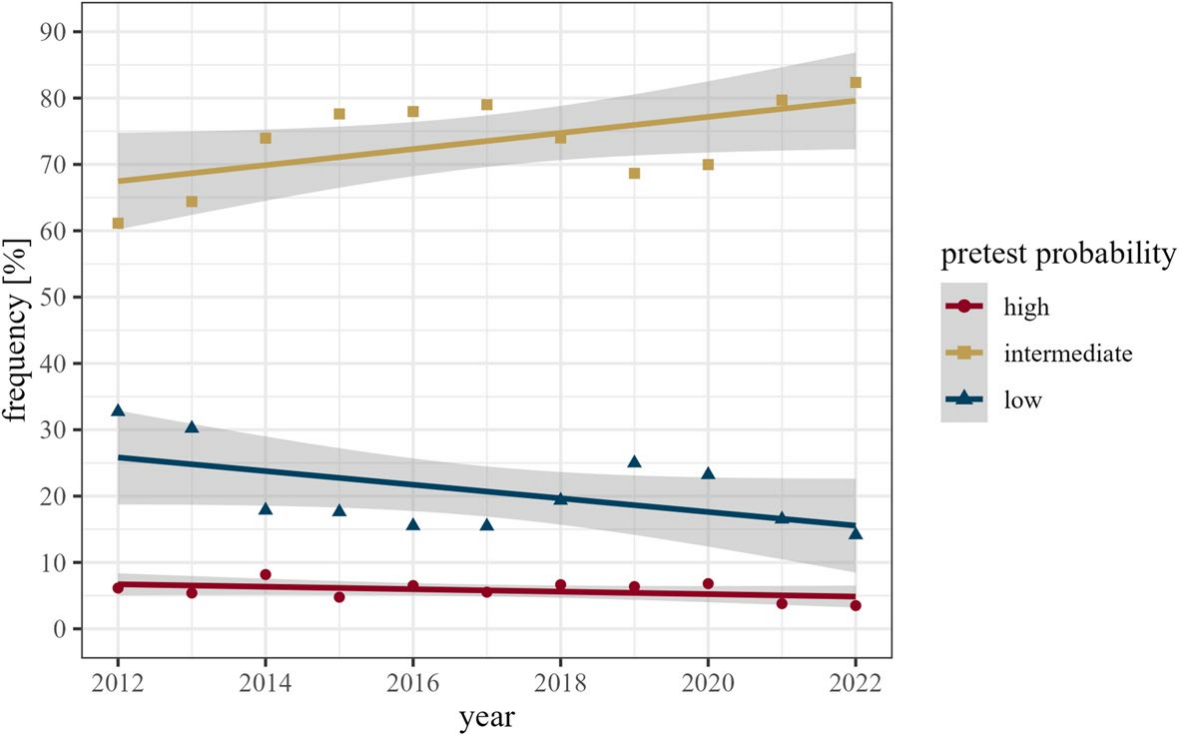
Distribution of pretest probabilities of CAD for annual cardiac CT submissions. Symbols depict data points. Lines represent linear regression models representing trends. Grey shading represents 95% confidence interval derived from the linear models

The principal indication for cardiac CT was suspected CAD (*n* = 121,051, 58.8%). TAVR planning was the indication in 20.9% (*n* = 43,141). Known valvular heart disease (*n* = 15,289, 7.4%), visualisation of pulmonary veins pre- or post-ablation (*n* = 13,354, 6.5%), and imaging of known CAD (*n* = 12,819, 6.2%) were other common indications. The top 10 indications of cardiac CT are listed in Table 1. The primary indication of suspected CAD showed a considerable proportional increase from 2012 to 2022 from 48.2 to 60.2% of all CT submissions (Fig. 4). Analysis of the pretest probabilities for CAD showed a decrease for the low pretest probability category, from 32.7% (905/2766) in 2012 to 14.1% (1063/7515) in 2022, and a corresponding increase in the intermediate pretest category from 61.1% (1691/2766) in 2012 to 82.4% (6189/7515) in 2022. CT for suspicion of CAD was only limitedly used in patients with a high pretest probability; they comprised 6.1% (170/2766) in 2012 and 3.5% (263/7515) in 2022 of all patients undergoing CT for CAD evaluation (Fig. 5).

**Table 1:** Top 10 indications for cardiac CT

Indication	Number of cases *N* = 205,999	Frequency
Suspected CAD	121,051	58.8%
TAVR planning	43,141	20.9%
Rule-out CAD in known heart valve disease	15,289	7.4%
Visualisation of pulmonary veins	13,354	6.5%
Known CAD	12,819	6.2%
Visualisation of coronary veins	3672	1.8%
Triple rule-out	3378	1.6%
Suspected valve disease	2043	1.0%
CABG-patency	1672	0.8%
Post-TAVR imaging	1651	0.8%

*CAD* coronary artery disease, *TAVR* transcatheter aortic valve replacement, *CABG* coronary artery bypass graft. *N* number of patients with cardiac CT

Categories are not mutually exclusive due to overlapping indications

The main indications for cardiac MRI examinations were suspected myocarditis (25.7%) and suspicion of CAD (21.4%), followed by suspected cardiomyopathy (19.3%), known CAD (16.2%), and myocardial viability (8.9%). The top 10 indications for cardiac MRI are listed in Table 2. The primary indication of suspected myocarditis showed a considerable annual increase from 2012 to 2022 from 23.4 to 36.9% of all MRI indications (Fig. 4).

**Table 2:** Top 10 indications for cardiac MRI

Indication	Number of cases *N* = 226,266	Frequency
Suspected myocarditis	58,260	25.7%
Suspected CAD	48,406	21.4%
Suspected cardiomyopathy	43,733	19.3%
Known CAD	36,615	16.2%
Viability assessment	20,079	8.9%
Known valve disease	7324	3.2%
Known congenital heart disease	6992	3.1%
Known cardiomyopathy	5596	2.5%
Suspected valve disease	4278	1.9%
Visualisation of pulmonary veins	4254	1.9%

*MRI* magnetic resonance imaging [examination], *CAD* coronary artery disease

*N* number of patients with cardiac MRI. Categories are not mutually exclusive due to overlapping indications

### Medication used for cardiac CT and MRI

The most common medication in cardiac CT was negative chronotropic medication for heart rate control such as beta-blockers (*n* = 49,766/205,999, 24.2%), ivabradine (*n* = 1097, 0.5%), and in rare cases (*n* = 19, 0.01%) calcium channel antagonists. The second most commonly used medications in CT were nitrates (*n* = 49,471, 24.0%) for vasodilation of the coronary arteries. Drugs for sedation (*n* = 556, 0.3%), premedication for known or suspected contrast media allergy (*n* = 416, 0.2%), or perchlorates for hyperthyroidism (*n* = 62, 0.03%) were also utilized, but rarely. In patients undergoing CT for suspected CAD, beta-blockers and nitrates were administered in 37.2% (*n* = 45,066/121,051) and 37.0% (*n* = 44,791/121,051) of examinations, respectively. More details on medication used for CT can be found in Table 3.

**Table 3:** Medication for cardiac CT

Medication	Number of cases in total *N* = 205,999	Frequency in total	Number of cases in suspected CAD *N* = 121,051	Frequency of cases in suspected CAD
Nitrates	49,471	24.0%	44,791	37.0%
Beta-blockers—oral	25,297	12.3%	23,087	19.1%
Beta-blockers—intravenous	24,469	11.9%	21,979	18.2%
Ivabradine	1097	0.5%	1066	0.9%
Sedation	556	0.3%	479	0.4%
Premedication for contrast allergy	416	0.2%	264	0.2%
Sodium perchlorate	62	0.03%	24	0.02%
Calcium channel blocker	19	0.01%	17	0.01%
Adenosine for myocardial perfusion study	15	0.01%	9	0.01%

*CAD* coronary artery disease. *N* number of patients in category

Medications are not mutually exclusive due to different indications

In cardiac MRI, pharmacological stress testing was conducted in 57,428 of 226,266 cases (25.4%). Stress imaging was mostly performed using adenosine (*n* = 50,131, 87.3%) or regadenoson (*n* = 6384, 11.1%) as stressor agent, whereas dobutamine was used only in 1.6% of cases (*n* = 913). In MRI for suspected CAD, stress testing was conducted more frequently than on average (*n* = 35,173/48,406, 72.7%). Other medication was used rarely, including sedating medication (483, 0.2%), nitrates (254, 0.1%), and negative chronotropic agents (*n* = 201, 0.1%). More details on medication in MRI can be found in Table 4.

**Table 4:** Medication for cardiac MRI

Medication	Number of cases in total *N* = 226,266	Frequency in total	Number of cases in suspected CAD *N* = 48,406	Frequency of cases in suspected CAD
Adenosine	50,131	22.2%	30,470	62.9%
Regadenoson	6384	2.8%	4106	8.5%
Dobutamine	913	0.4%	597	1.2%
Sedation	483	0.2%	107	0.2%
Nitrates	254	0.1%	145	0.3%
Beta-blockers—intravenous	108	0.05%	56	0.1%
Beta-blockers—oral	85	0.04%	23	0.05%
Premedication for contrast allergy	46	0.02%	11	0.02%
Calcium channel blocker	8	0.004%	4	0.01%

*CAD* coronary artery disease. *N* number of patients in category

Medications are not mutually exclusive due to different indications

### Safety evaluation

In both MRI and CT, the rate of adverse events was very low. Safety assessment for CT revealed adverse events in 0.3% of cases (*n* = 547/205,999), of which 0.17% were related to a hypersensitivity reaction to iodinated contrast media (*n* = 366/205,999). Extravasation of contrast media accounted for another 0.08% of adverse events (*n* = 172/205,999). Additionally, six cases of contrast-induced nephropathy (0.003%, *n* = 6/205,999) and two instances of thyrotoxic crisis (0.001%, *n* = 2/205,999) were reported. The six events of contrast-induced nephropathy occurred in five male patients and one female patient (median age 81.5 years, IQR 4.75 years); indications for these studies were workup for TAVR in two, suspected CAD in two, and triple rule-out in two patients.

In MRI, adverse events were reported in 0.7% of cases (*n* = 1649/226,266) and occurred more often in stress (0.8%, *n* = 458/57,428) as compared to non-stress (0.7%, *n* = 1191/168,838) examinations (*p* = 0.027). The most frequently reported adverse events were dyspnoea (*n* = 501/226,266, 0.2%) followed by hypersensitivity reactions to contrast media (*n* = 267/226,266, 0.12%). Very rare occurrences included the accidental discovery of an implanted pacemaker or ICD device (*n* = 12/226,266, 0.005%) and contrast-induced nephropathy (*n* = 3/226,266, 0.001%).

### Reporting approaches

**Fig. 6:**
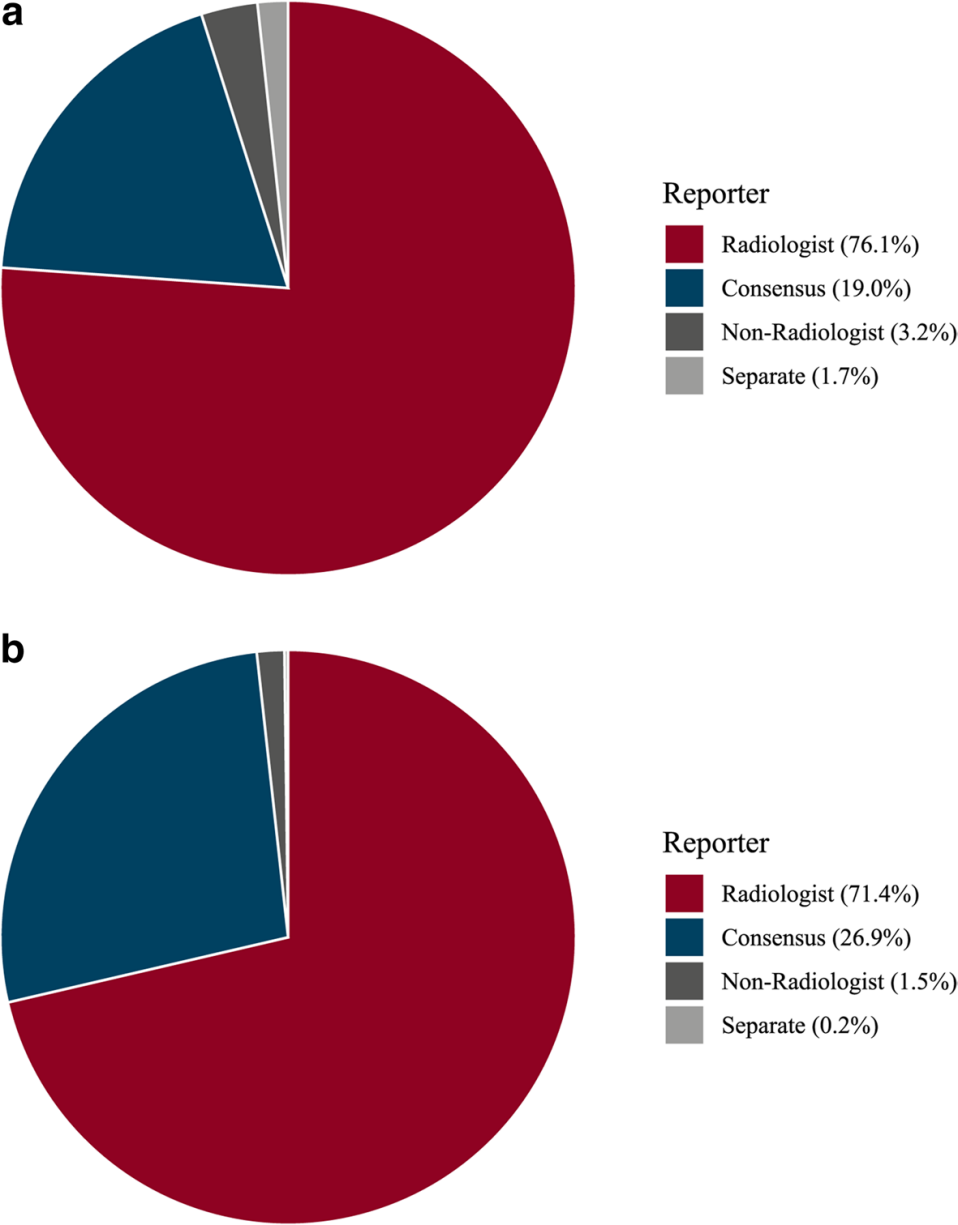
Reporting practices based on MRCT-registry data for CT (**a**) and MRI (**b**). Consensus: Examination read by radiologist and cardiologist in consensus

Most submitted cases were reported by radiologists alone (76.1% for CT, 71.4% for MRI), followed by joint consensus readings of radiologists with non-radiologists (19.0% and 26.9%, respectively). The remaining 4.9% (CT) and 1.7% (MRI) examinations were reported by non-radiological specialties, or in separate readings of radiologists and non-radiologists. A graphical representation of the reporting statistics is shown in Fig. 6.

## Discussion

The use of cardiac CT and MRI is poised for further growth, based on several scientific publications, guidelines, and recommendation papers by medical societies in Europe and elsewhere [7, 15–18, 27–29]. Therefore, more radiologists trained in multimodal cardiac imaging are required to help cover this demand. In this regard, the current study confirms and expands on the previous literature as follows: (*i*) the MRCT-registry is the largest available radiological database including more than 400,000 entries on CT and MRI examinations; (*ii*) The MRCT-registry is fed by the majority of European countries (total 29) and a few non-European countries; (*iii*) the MRCT-registry demonstrates a continuous increase in the number of performed cardiac CT and MRI examinations across Europe; (*iv*) the growth in the number of examinations was most pronounced for the evaluation of CAD with CT and for the evaluation of myocarditis with MRI, in line with current guidelines and recommendations; (*v*) both cardiac CT and MRI examinations, although many require the application of medication, are safe imaging procedures with very low rates for adverse events; and (*vi*) most submitted cardiac CT (76%) and MRI (71%) examinations were read exclusively by radiologists.

The growing demand for multimodality cardiac imaging expertise [30–32] not only poses significant challenges regarding the expected increasing workload, scanner capacity, and required trained personnel. It also implies the necessity for a centralized, well-defined pathway of access to cardiac CT and MR imaging services, managed by an independent, objective, and well-trained imaging professional, hereby balancing clinical question with available equipment and economic resources in an increasingly challenging healthcare landscape. The MRCT-registry, the largest of its kind, stands now as a testament to the prominent role that radiologists have assumed, not only in providing these necessary imaging services, but also in guiding their correct implementation in the patient’s best interest.

A crucial point to further note, as also shown in a previous sub-analysis of the MRCT-registry data, is that cardiac CT and MRI examinations have disseminated beyond academic centres to additionally include non-academic hospitals and private practices, increasing their accessibility to broader patient cohorts (for illustration, see Fig. 3) [21–23].

The observed rise in examinations most probably indicates appropriate referral, as indications for both CT and MR are firmly aligned with the most recent guidelines [7, 15, 16, 27–29]. CT angiography for patients with suspected CAD is primarily used in low and intermediate pretest scenarios [7]. Adherence to these recommendations is evident in the MRCT-registry analysing the data on CT-based assessment of CAD showing a continuous increase in patients at intermediate pretest probability. The rise in MRI-based assessment of suspected or known myocarditis is also well supported by current literature [33].

Finally, the MRCT data confirms that cardiac CT and MRI procedures, despite frequently necessitating cardiac medication, are safe examinations with only a minimal probability of adverse events. The reported very low adverse rates (0.3% for CT and 0.7% for MRI) are in line with previous literature [24] and randomized trials [34].

The presented data unequivocally reinforces the fundamental premise that radiologists, through their multimodality-based training with a profound understanding of indications, state-of-the-art technology, and imaging findings in and around the organ of interest, are in an ideal position to face future challenges and ensure the correct application and expansion of cardiac CT/MRI services. The European Society of Radiology (ESR) has, being fully aware of this responsibility, established a multilevel, standardized educational framework for imaging, as detailed in the European Training Curriculum (https://www.myesr.org/education/training-curricula/). This curriculum formally includes cardiac multimodality imaging as a core component of every radiology residency, ensuring that at the end of their training, all residents are equally competent to perform and interpret cardiac CT and MR examinations. It also ensures that radiologist not only adeptly manage modern and evolving imaging technologies, but, through their broad imaging training, also accurately diagnose incidental or concomitant diseases beyond cardiac pathology [35], providing as such a complete assessment of all available image data across different organ systems. This competency becomes increasingly important as the need for, e.g., cardiovascular CT imaging expands to emergency departments where the availability of around-the-clock services is essential to assess the various cardiac and non-cardiac differential diagnoses of acute chest pain [36]. Moreover, given that newer imaging technology with fast volume coverage further improves the depiction of the heart even in non-gated chest examinations hereby potentially improving risk stratification and diagnosis [37], it can be assumed that knowledge about cardiac imaging findings will become increasingly relevant for every radiologist in the near future.

While the general growth in medical imaging over the past decades yields unarguable benefits to patients in terms of longer and higher quality of life [38], part of the growth in imaging utilization could also be attributed to overutilization. Inadequate use of imaging resources is a well-known and multifactorial problem. Prior research has explored various factors contributing to overutilization, notably including payment structures, financial incentives, and self-referral, with the latter being particularly significant [39]. Self-referral is the act of a physician referring a patient to him- or herself for additional diagnostic or therapeutic procedures, which can result in financial profit. This poses a concern not just economically, but also in terms of increased exposure to ionizing radiation for both individuals and the general population [40]. Radiology, by definition, is a profession that works on referral from other medical disciplines and, as such, is not susceptible to self-referral. This eliminates conflicts of interests, allowing radiologists to collaboratively determine the most suitable imaging method with the referring physician, based on the patient’s specific clinical needs. This approach also underscores the role of radiologists as a gatekeeper for a correct value-based use of healthcare resources.

This study has the following limitations. First, the results of this study are based on registry data and may be susceptible to reporting bias. Second, no follow-up data regarding patient outcome or late-onset adverse events was available. Third, detailed information about the individual contributing centres in the MRCT-registry is missing. Thus, in-depth analyses on the reasons of increasing case submissions to the registry are not possible. Finally, some parameters which could be used to further analyse the use of cardiac CT and MRI are not recorded in the registry, such as the setting of the scan (outpatient, inpatient, emergency department).

In conclusion, real-life data on cardiac imaging in Europe using the MRCT-registry demonstrates a considerable increase in examinations over the past years, the vast majority of which are read by radiologists. Our results suggest that radiologists have an increasingly important role to provide cardiac CT and MR imaging services, contributing to expanding availability and expertise in both academic and non-academic centres. Radiology acts as a crucial barrier against the overutilization of medical imaging, ensuring correct application of different modalities according to current recommendations and guidelines.

### Supplementary Information

Below is the link to the electronic supplementary material.

Supplementary file1 (PDF 156 KB)

## Abbreviations

CAD: Coronary artery disease
DRG: German Roentgen Society
EBCR: European Board of Cardiovascular Radiology
ESCR: European Society of Cardiovascular Radiology
ESR: European Society of Radiology
TAVR: Transcatheter aortic valve replacement

## Declarations

### Guarantor

The scientific guarantor of this publication is H. Alkadhi.

### Statistics and biometry

One of the authors has statistical expertise.

No complex statistical methods were necessary for this paper.

### Study subjects or cohorts overlap

Several earlier papers have been published using data from the MRCT-registry, such as Foldyna B et al (2022) Quality and safety of coronary computed tomography angiography at academic and non-academic sites: insights from a large European registry (ESCR MR/CT Registry). *Eur Radiol* 32:5246–5255, but with other patient populations from the registry and with other purposes.

### Methodology

• retrospective

• registry data

## References

[1] Douglas PS, Hoffmann U, Patel MR et al (2015) Outcomes of anatomical versus functional testing for coronary artery disease. N Engl J Med 372:1291–1300 25773919 10.1056/NEJMoa1415516 PMC4473773

[2] Group DT, Maurovich-Horvat P, Bosserdt M et al (2022) CT or invasive coronary angiography in stable chest pain. N Engl J Med 386:1591–1602 35240010 10.1056/NEJMoa2200963

[3] Investigators S-H, Newby DE, Adamson PD et al (2018) Coronary CT angiography and 5-year risk of myocardial infarction. N Engl J Med 379:924–933 30145934 10.1056/NEJMoa1805971

[4] Nagel E, Greenwood JP, McCann GP et al (2019) Magnetic resonance perfusion or fractional flow reserve in coronary disease. N Engl J Med 380:2418–2428 31216398 10.1056/NEJMoa1716734

[5] Greenwood JP, Maredia N, Younger JF et al (2012) Cardiovascular magnetic resonance and single-photon emission computed tomography for diagnosis of coronary heart disease (CE-MARC): a prospective trial. Lancet 379:453–460 22196944 10.1016/S0140-6736(11)61335-4 PMC3273722

[6] Mezquita AJV, Biavati F, Falk V et al (2023) Clinical quantitative coronary artery stenosis and coronary atherosclerosis imaging: a Consensus Statement from the Quantitative Cardiovascular Imaging Study Group. Nat Rev Cardiol 20:696–714 37277608 10.1038/s41569-023-00880-4

[7] Knuuti J, Wijns W, Saraste A et al (2020) 2019 ESC Guidelines for the diagnosis and management of chronic coronary syndromes. Eur Heart J 41:407–477 31504439 10.1093/eurheartj/ehz425

[8] Francone M, Budde RPJ, Bremerich J et al (2020) CT and MR imaging prior to transcatheter aortic valve implantation: standardisation of scanning protocols, measurements and reporting-a consensus document by the European Society of Cardiovascular Radiology (ESCR). Eur Radiol 30:2627–2650 31489471 10.1007/s00330-019-06357-8 PMC7160220

[9] Ge Y, Gupta S, Fentanes E et al (2021) Role of cardiac CT in pre-procedure planning for transcatheter mitral valve replacement. JACC Cardiovasc Imaging 14:1571–1580 33865768 10.1016/j.jcmg.2020.12.018

[10] Andreini D, Collet C, Leipsic J et al (2022) Pre-procedural planning of coronary revascularization by cardiac computed tomography: an expert consensus document of the Society of Cardiovascular Computed Tomography. EuroIntervention 18:e872–e887 35994043 10.4244/EIJ-E-22-00036 PMC9743242

[11] Schwitter J, Wacker CM, Wilke N et al (2013) MR-IMPACT II: Magnetic Resonance Imaging for Myocardial Perfusion Assessment in Coronary artery disease Trial: perfusion-cardiac magnetic resonance vs. single-photon emission computed tomography for the detection of coronary artery disease: a comparative multicentre, multivendor trial. Eur Heart J 34:775–781 22390914 10.1093/eurheartj/ehs022

[12] Arai AE, Schulz-Menger J, Shah DJ et al (2023) Stress perfusion cardiac magnetic resonance vs SPECT imaging for detection of coronary artery disease. J Am Coll Cardiol 82:1828–1838 37914512 10.1016/j.jacc.2023.08.046

[13] McDonagh TA, Metra M, Adamo M et al (2021) 2021 ESC Guidelines for the diagnosis and treatment of acute and chronic heart failure. Eur Heart J 42:3599–3726 34447992 10.1093/eurheartj/ehab368

[14] Baumgartner H, De Backer J, Babu-Narayan SV et al (2021) 2020 ESC Guidelines for the management of adult congenital heart disease. Eur Heart J 42:563–645 32860028 10.1093/eurheartj/ehaa554

[15] Arbelo E, Protonotarios A, Gimeno JR et al (2023) 2023 ESC Guidelines for the management of cardiomyopathies. Eur Heart J 44:3503–3626 37622657 10.1093/eurheartj/ehad194

[16] Esposito A, Gallone G, Palmisano A, Marchitelli L, Catapano F, Francone M (2020) The current landscape of imaging recommendations in cardiovascular clinical guidelines: toward an imaging-guided precision medicine. Radiol Med 125:1013–1023 32964326 10.1007/s11547-020-01286-9 PMC7593299

[17] Dreisbach JG, Nicol ED, Roobottom CA, Padley S, Roditi G (2018) Challenges in delivering computed tomography coronary angiography as the first-line test for stable chest pain. Heart (British Cardiac Society) 104:921–927 29138258 10.1136/heartjnl-2017-311846 PMC5969350

[18] van den Boogert TPW, Claessen B, Boekholdt SM et al (2021) The impact and challenges of implementing CTCA according to the 2019 ESC guidelines on chronic coronary syndromes: a survey and projection of CTCA services in the Netherlands. Insights Imaging 12:186 34921633 10.1186/s13244-021-01122-2 PMC8684565

[19] Natale L, Vliegenthart R, Salgado R et al (2023) Cardiac radiology in Europe: status and vision by the European Society of Cardiovascular Radiology (ESCR) and the European Society of Radiology (ESR). Eur Radiol 33:5489–5497 36905466 10.1007/s00330-023-09533-z PMC10006558

[20] Gatti M, Liguori C, Muscogiuri G et al (2021) Challenges and opportunities to delivering cardiac imaging training: a national survey by the Italian college of cardiac radiology. Insights Imaging 12:136 34570297 10.1186/s13244-021-01076-5 PMC8475361

[21] Langenbach MC, Sandstede J, Sieren MM et al (2023) German Radiological Society and the Professional Association of German Radiologists Position Paper on coronary computed tomography: clinical evidence and quality of patient care in chronic coronary syndrome. Rofo 195:115–134 36634682 10.1055/a-1973-9687

[22] Sieren MM, Maintz D, Gutberlet M et al (2022) Current status of cardiovascular imaging in Germany: structured data from the National Certification Program, ESCR Registry, and Survey among Radiologists. Rofo 194:181–191 34384112 10.1055/a-1554-9236

[23] Foldyna B, Uhlig J, Gohmann R et al (2022) Quality and safety of coronary computed tomography angiography at academic and non-academic sites: insights from a large European registry (ESCR MR/CT Registry). Eur Radiol 32:5246–5255 35267087 10.1007/s00330-022-08639-0

[24] Uhlig J, Lucke C, Vliegenthart R et al (2019) Acute adverse events in cardiac MR imaging with gadolinium-based contrast agents: results from the European Society of Cardiovascular Radiology (ESCR) MRCT Registry in 72,839 patients. Eur Radiol 29:3686–3695 31041566 10.1007/s00330-019-06171-2 PMC6554260

[25] Uhlig J, Al-Bourini O, Salgado R et al (2020) Gadolinium-based contrast agents for cardiac MRI: use of linear and macrocyclic agents with associated safety profile from 154 779 European patients. Radiol Cardiothorac Imaging 2:e200102 33778622 10.1148/ryct.2020200102 PMC7977928

[26] Taylor AJ, Cerqueira M, Hodgson JM et al (2010) ACCF/SCCT/ACR/AHA/ASE/ASNC/NASCI/SCAI/SCMR 2010 appropriate use criteria for cardiac computed tomography. A report of the American College of Cardiology Foundation Appropriate Use Criteria Task Force, the Society of Cardiovascular Computed Tomography, the American College of Radiology, the American Heart Association, the American Society of Echocardiography, the American Society of Nuclear Cardiology, the North American Society for Cardiovascular Imaging, the Society for Cardiovascular Angiography and Interventions, and the Society for Cardiovascular Magnetic Resonance. J Am Coll Cardiol 56:1864–1894 21087721 10.1016/j.jacc.2010.07.005

[27] Albus C, Barkhausen J, Fleck E, Haasenritter J, Lindner O, Silber S (2017) The diagnosis of chronic coronary heart disease. Dtsch Arztebl Int 114:712–719 29122104 10.3238/arztebl.2017.0712 PMC5686296

[28] Cury RC, Leipsic J, Abbara S et al (2022) CAD-RADS 2.0 - 2022 Coronary Artery Disease-Reporting and Data System: an expert consensus document of the Society of Cardiovascular Computed Tomography (SCCT), the American College of Cardiology (ACC), the American College of Radiology (ACR), and the North America Society of Cardiovascular Imaging (NASCI). JACC Cardiovasc Imaging 15:1974–2001 36115815 10.1016/j.jcmg.2022.07.002

[29] Pontone G, Di Cesare E, Castelletti S et al (2021) Appropriate use criteria for cardiovascular magnetic resonance imaging (CMR): SIC-SIRM position paper part 1 (ischemic and congenital heart diseases, cardio-oncology, cardiac masses and heart transplant). Radiol Med 126:365–379 33629237 10.1007/s11547-020-01332-6 PMC7937599

[30] Weir-McCall JR, Williams MC, Shah ASV et al (2023) National trends in coronary artery disease imaging: associations with health care outcomes and costs. JACC Cardiovasc Imaging 16:659–671 36752441 10.1016/j.jcmg.2022.10.022

[31] Reeves RA, Halpern EJ, Rao VM (2021) Cardiac imaging trends from 2010 to 2019 in the Medicare population. Radiol Cardiothorac Imaging 3:e210156 34778785 10.1148/ryct.2021210156 PMC8581585

[32] Goldfarb JW, Weber J (2021) Trends in cardiovascular MRI and CT in the U.S. Medicare population from 2012 to 2017. Radiol Cardiothorac Imaging 3:e200112 33778651 10.1148/ryct.2021200112 PMC7977977

[33] Caobelli F, Cabrero JB, Galea N et al (2023) Cardiovascular magnetic resonance (CMR) and positron emission tomography (PET) imaging in the diagnosis and follow-up of patients with acute myocarditis and chronic inflammatory cardiomyopathy: a review paper with practical recommendations on behalf of the European Society of Cardiovascular Radiology (ESCR). Int J Cardiovasc Imaging 39:2221–2235 37682416 10.1007/s10554-023-02927-6 PMC10674005

[34] Schonenberger E, Martus P, Bosserdt M et al (2019) Kidney injury after intravenous versus intra-arterial contrast agent in patients suspected of having coronary artery disease: a randomized trial. Radiology 292:664–672 31264950 10.1148/radiol.2019182220

[35] Williams MC, Hunter A, Shah ASV et al (2018) Impact of noncardiac findings in patients undergoing CT coronary angiography: a substudy of the Scottish computed tomography of the heart (SCOT-HEART) trial. Eur Radiol 28:2639–2646 29294153 10.1007/s00330-017-5181-5 PMC5938292

[36] Loewe C, Beitzke D, Francone M (2023) How to set up a 24/7 cardiac computed tomography service in an emergency department. Eur Radiol 33:8177–8179 37160425 10.1007/s00330-023-09721-x

[37] Xia C, Vonder M, Sidorenkov G et al (2021) Cardiovascular risk factors and coronary calcification in a middle-aged Dutch population: the ImaLife study. J Thorac Imaging 36:174–180 33060489 10.1097/RTI.0000000000000566 PMC8132906

[38] Hendrix W, Rutten M, Hendrix N et al (2023) Trends in the incidence of pulmonary nodules in chest computed tomography: 10-year results from two Dutch hospitals. Eur Radiol 33:8279–8288 37338552 10.1007/s00330-023-09826-3 PMC10598118

[39] Hendee WR, Becker GJ, Borgstede JP et al (2010) Addressing overutilization in medical imaging. Radiology 257:240–245 20736333 10.1148/radiol.10100063

[40] Thrall JH (2012) Radiation exposure in CT scanning and risk: where are we? Radiology 264:325–328 22821692 10.1148/radiol.12121137

