# Supplementary material for: Competence of radiologists in cardiac CT and MR imaging in Europe: insights from the ESCR Registry
Source: Eur Radiol. 2024 Feb 28;34(9):5666–77. doi: 10.1007/s00330-024-10644-4 (PMC11364788; doi:10.1007/s00330-024-10644-4)
Supplement: Supplementary file 1 — Supplementary file1 (PDF 156 KB) [file 330_2024_10644_MOESM1_ESM.pdf]

Competence of Radiologists in Cardiac CT and MR Imaging in Europe: Insights from the ESCR Registry

ELECTRONIC SUPPLEMENTARY MATERIAL

Table S1: Data collected in ESCR registry for CT and MRI

**CT**	**MRI**
Date of Examination	Date of Examination
Age	Age
Gender	Gender
Body Weight	Body Weight
Height	Height
**Indications**	
Suspected CAD	Suspected CAD
Known CAD	Known CAD
Known CAD/single vessel disease	Known CAD/single vessel disease
Known CAD/multi vessel disease	Known CAD/multi vessel disease
Known CAD/Hx of myocardial infarction	Known CAD/Hx of myocardial infarction
Known CAD/Hx of Cardiac Catheterization	Known CAD/Hx of Cardiac Catheterization
Known CAD/Hx of CABG	Known CAD/Hx of CABG
Known CAD/Hx of PCI or Stenting	Known CAD/Hx of PCI or Stenting
Suspected HTx Rejection	Suspected HTx Rejection
Suspected HTx Allograft Vasculopathy	Suspected HTx Allograft Vasculopathy
Visualisation of Pulmonary Veins	Visualisation of Pulmonary Veins
Visualisation of Coronary Veins	Visualisation of Coronary Veins
Diagnosis/Coronary Anomaly or Fistula	Diagnosis/Coronary Anomaly or Fistula
Diagnosis/CABG or IMA-Graft-Patency	Diagnosis/CABG or IMA-Graft-Patency
Diagnosis/Pericardial Disease	Diagnosis/Pericardial Disease
Diagnosis/Triple-Rule-Out	
Diagnosis/Viability Assessment	Diagnosis/Viability Assessment
Diagnosis/Pre-TAVI	Diagnosis/Pre-TAVI
Diagnosis/Post-TAVI	Diagnosis/Post-TAVI
Suspected Cardiomyopathy	Suspected Cardiomyopathy
Known Cardiomyopathy	Known Cardiomyopathy
Suspected Myocarditis	Suspected Myocarditis
Known Myocarditis	Known Myocarditis
Suspected Valve Disease	Suspected Valve Disease
Known Valve Disease	Known Valve Disease
Suspected Cardiac Tumor	Suspected Cardiac Tumour
Known Cardiac Tumor	Known Cardiac Tumour
Suspected Congenital Heart Disease (CHD)	Suspected Congenital Heart Disease (CHD)
Other Suspected Congenital Heart Disease	Other Suspected Congenital Heart Disease
Known Congenital Heart Disease (CHD)/	Known Congenital Heart Disease (CHD)/
Other Known Congenital Heart Disease	Other Known Congenital Heart Disease
Others Indications	Other Indications

**Adverse Events**	
Complications - Adverse Events (AE)	Complications - Adverse Events (AE)
Complications - Adverse Events (AE)/extravasation injury	Complications - Adverse Events (AE)/extravasation injury
Complications - Adverse Events (AE)/hypersensitive reaction to contrast ...	Complications - Adverse Events (AE)/hypersensitive reaction to contrast ...
Complications - Adverse Events (AE)/dyspnoea	Complications - Adverse Events (AE)/dyspnoea
Complications - Adverse Events (AE)/pulmonary oedema	Complications - Adverse Events (AE)/pulmonary oedema
Complications - Adverse Events (AE)/severe allergic reaction	Complications - Adverse Events (AE)/severe allergic reaction
Complications - Adverse Events (AE)/symptomatic bradycardia	Complications - Adverse Events (AE)/symptomatic bradycardia
Complications - Adverse Events (AE)/symptomatic hypotension	Complications - Adverse Events (AE)/symptomatic hypotension
Complications - Adverse Events (AE)/renal failure	Complications - Adverse Events (AE)/renal failure
Complications - Adverse Events (AE)/thyreotoxicosis	Complications - Adverse Events (AE)/thyreotoxicosis
Complications - Adverse Events (AE)/resuscitation	Complications - Adverse Events (AE)/resuscitation
Complications - Adverse Events (AE)/heating	Complications - Adverse Events (AE)/heating
Complications - Adverse Events (AE)/accidentially Pacemaker/ICD detected	Complications - Adverse Events (AE)/accidentially Pacemaker/ICD detected
Complications - Adverse Events (AE)/other	Complications - Adverse Events (AE)/Others
Other Complications - Adverse Events	Other Complications - Adverse Events
**Premedication**	
Premedication for/during CT	Premedication for/during MR
Premedication for/during CT/sedation	Premedication for/during MR/sedation
Premedication for/during CT/nitrates	Premedication for/during MR/nitrates
Premedication for/during CT/beta-blocker i.v.	Premedication for/during MR/beta-blocker i.v.
Premedication for/during CT/beta-blocker orally	Premedication for/during MR/beta-blocker orally
Premedication for/during CT/ivabradine	Premedication for/during MR/ivabradine
Premedication for/during CT/Ca-channel blocker	Premedication for/during MR/Ca-channel blocker
Premedication for/during CT/premedication for contrast allergy	Premedication for/during MR/premedication for contrast allergy
Premedication for/during CT/adenosine for perfusion study	MR-Perfusionstudy (Stress-Adenosine)
	MR-Perfusionstudy (Stress-Regadenoson)
Premedication for/during CT/dobutamine for perfusion study	MR-Perfusionstudy (Stress-Dobutamin)
Premedication for/during CT/sodiumperchlorate	

Premedication for/during CT/other	Premedication for/during MR/others
Mean Heart Rate	Mean Heart Rate
Heart Rhythm (during scan)	Heart Rhythm (during scan)
Other Heart Rhythm (during scan)	Other Heart Rhythm (during scan)
**Reporting**	
Reporter	Reporter
Other Reporter	Other Reporter
Role	Role
